# A Systematic Review and Meta-Analysis Exploring Variations in Copper Levels between Individuals with Malaria and Uninfected Controls

**DOI:** 10.3390/nu15224749

**Published:** 2023-11-10

**Authors:** Kwuntida Uthaisar Kotepui, Aongart Mahittikorn, Polrat Wilairatana, Frederick Ramirez Masangkay, Manas Kotepui

**Affiliations:** 1 Medical Technology Program, School of Allied Health Sciences, Walailak University, Tha Sala, Nakhon Si Thammarat 80160, Thailand; kwuntida.ut@wu.ac.th; 2 Department of Protozoology, Faculty of Tropical Medicine, Mahidol University, Bangkok 10400, Thailand; aongart.mah@mahidol.ac.th; 3 Department of Clinical Tropical Medicine, Faculty of Tropical Medicine, Mahidol University, Bangkok 10400, Thailand; 4 Department of Medical Technology, Faculty of Pharmacy, University of Santo Tomas, Manila 1008, Philippines

**Keywords:** malaria, *Plasmodium*, systematic review, copper, micronutrients, meta-analysis

## Abstract

Micronutrient insufficiency has been implicated in malaria pathogenesis. However, the role of copper in malaria remains inconclusive. This study aimed to investigate the association between copper levels and malaria pathogenesis, providing a deeper understanding of copper’s role in the disease. A systematic review was conducted following the registered protocol in PROSPERO (CRD42023439732). Multiple databases, including Embase, MEDLINE, Ovid, PubMed, Scopus, and Google Scholar, were searched for relevant studies reporting blood copper levels in patients with malaria. The Joanna Briggs Institute critical appraisal checklist was used for assessing methodological quality. Qualitative and quantitative syntheses were employed, organizing, and summarizing the findings of the included studies. To calculate the standardized mean difference (Hedge’s g) and 95% confidence intervals (CIs), a random-effects model was used. After screening the databases, 16 studies were included. Most studies (52.9%) reported that individuals with malaria had significantly higher copper levels than uninfected controls. The meta-analysis, based on 16 studies, showed no significant difference in copper levels between patients with malaria and uninfected controls overall (*p* = 0.39; Hedges’ g, 0.38; 95% CI, −0.48 to 1.25; *I*^2^, 98.73%). Subgroup analysis showed a significant difference in copper levels between patients with malaria and uninfected controls among studies conducted in Asia (*p* < 0.01; Hedges’ g, 1.74; 95% CI, 1.04 to 2.44; *I*^2^, 90.88%; five studies) and studies employing plasma blood samples (*p* < 0.01; Hedges’ g, 1.13; 95% CI, 0.60 to 2.07; *I*^2^, 93.11%; four studies). The qualitative synthesis of the reviewed studies suggests a complex relationship between copper levels and malaria. The meta-analysis results showed no significant difference in copper levels between patients with malaria and uninfected controls overall. However, subgroup analyses based on various factors, including continent and blood sample type, showed copper level variations. These findings highlight the need for further research to better understand the role of copper in malaria pathogenesis by considering geographical factors and the blood sample type used for copper level measurement.

## 1. Introduction

The global prevalence of malaria remains a significant public health concern, with the highest disease burden observed in regions such as Sub-Saharan Africa [1]. According to the World Malaria Report 2022, there were approximately 247 million cases and 619,000 deaths worldwide in 2021 [1]. Malaria is caused by infection with *Plasmodium* species, which are parasitic protozoan organisms transmitted to humans through the bites of infected female *Anopheles* mosquitoes. *Plasmodium falciparum* (*P. falciparum*), *Plasmodium vivax* (*P. vivax*), *Plasmodium malariae* (*P. malariae*), *Plasmodium ovale* (*P. ovale*), and *Plasmodium knowlesi* (*P. knowlesi*) are the five main *Plasmodium* species causing malaria in humans [2]. *P. falciparum* is responsible for most malaria-related deaths, particularly in Sub-Saharan Africa, and is associated with severe complications. Conversely, *P. vivax* has a broader distribution and can cause recurring malaria episodes as it can lie dormant in the liver [3]. Although less common, *P. malariae* and *P. ovale* can still lead to persistent infections and relapses [4,5]. Additionally, *P. knowlesi*, traditionally considered a monkey malaria parasite, can infect humans and cause severe malaria, primarily in Southeast Asia [6]. Malaria can be diagnosed by observing parasites in a blood smear using light microscopy as the gold standard method. Rapid diagnostic tests (RDTs), immunofluorescence, and molecular techniques are other detection methods [7,8]. Moreover, newer techniques, including the fluorescent Blue-ray optical device [9], magneto-optical diagnosis [10], and micromagnetic resonance relaxometry [11], offer the rapid, sensitive, and quantitative detection of malaria parasites.

Micronutrient insufficiency, including deficiencies of iron, zinc, vitamin A, and other micronutrients, has been reported to be associated with malaria pathogenesis [12,13,14]. Supplementation of these micronutrients has been proposed as a preventive intervention for reducing malaria prevalence, severity, or deaths [15,16,17]. Copper, an essential trace element, plays a crucial role as a catalyst in heme synthesis and iron absorption [18]. It is involved in various biological processes, including red blood cell formation, iron metabolism, and immune system stimulation, which aids in combating infections, facilitating tissue repair, and promoting healing [19]. Furthermore, copper contributes to stimulating the immune system, thereby enabling it to combat infections, facilitate tissue repair, and promote healing [20,21]. However, an in vitro study has provided strong evidence suggesting that copper can induce oxidative damage in erythrocytes and enhance reactive oxygen and nitrogen species generation [22].

Considering the inconsistent findings regarding the role of copper in malaria pathogenesis, investigating copper levels in individuals with *Plasmodium* infection is crucial. Therefore, this study aimed to investigate the association between copper levels and malaria pathogenesis, aiming to enhance our understanding of the role of copper in the pathogenesis of malaria. The results of this study will contribute to bridging the knowledge gap in this area. Moreover, gaining insights into the role of copper in malaria pathogenesis may have implications for managing patients with malaria, potentially including the consideration of copper supplementation as a therapeutic approach.

## 2. Materials and Methods

### 2.1. International Prospective Register of Systematic Review (PROSPERO) Registration

The systematic review protocol was registered with the PROSPERO number CRD42023439732. This systematic review was reported according to the updated guidelines for reporting systematic reviews, the Preferred Reporting Items for Systematic reviews and Meta-Analyses (PRISMA) 2020 statement [23].

### 2.2. Outcomes

The main outcome of this systematic review was the difference in blood copper levels between individuals with malaria and uninfected controls. The secondary outcome focused on blood copper level variations in relation to parasite density.

### 2.3. Search Strategy

A comprehensive literature search was conducted using the Embase, MEDLINE, Ovid, PubMed, and Scopus databases to identify relevant studies published until June 2023. The search was designed to be as inclusive as possible to capture studies that investigated the relationship between copper levels and malaria. The following combination of Medical Subject Heading (MeSH) terms and keywords such as “copper” and “malaria” was employed: “copper AND (malaria” OR “*Plasmodium*” OR “*Plasmodium* Infection“ OR “Remittent Fever“ OR “Marsh Fever“ OR “Paludism)” (Table S1). Additionally, a search was conducted in Google Scholar; to identify any potential studies that might have been missed in the initial search, the reference lists of selected articles were reviewed.

### 2.4. Inclusion and Exclusion Criteria

Studies were included if they met the following criteria: (1) original research articles; (2) case–control, cohort, cross-sectional studies, or other designs; (3) assessed copper levels in humans with and without malaria; and (4) provided sufficient data for qualitative synthesis and effect size calculation. Exclusion criteria encompassed review articles, case reports, letters to editors, studies not conducted in humans, and studies that did not directly assess copper levels in relation to malaria.

### 2.5. Study Selection and Data Extraction

Two authors independently performed the study selection and data extraction. Initially, titles and abstracts of the articles retrieved through the search were screened to assess their relevance. At this stage, clearly irrelevant studies were excluded. Next, full texts of the articles that passed the initial screening were reviewed. At this stage, the articles were assessed against the inclusion and exclusion criteria. Those that did not meet these criteria were excluded, and the reason for exclusion was often documented. The extracted information included the first author’s name, publication year, study design, study location, participants’ age, assessment methods for copper levels and malaria parasites, parasite density, and types of blood samples for copper level measurement. Any discrepancies between the two authors were resolved through discussion or consultation with a third author if necessary.

### 2.6. Quality Assessment

The Joanna Briggs Institute (JBI) critical appraisal checklist for observational studies was used for assessing the methodological quality of the included studies [24]. For case–control studies, the tool enabled the evaluation of different quality aspects, including group comparability, the matching of cases and controls, exposure measurement, confounding factors, and statistical analysis. For cross-sectional studies, the JBI tool assesses the methodological quality of research by examining aspects, including inclusion criteria, sample representativeness, exposure measurement, confounding factor management, outcome measurement, and statistical analysis. Two authors independently performed quality assessment. Any discrepancies between reviewers were resolved through discussion or consultation with a third reviewer if necessary.

### 2.7. Data Synthesis and Analysis

A qualitative synthesis approach was employed, wherein the findings of the included studies were systematically organized and summarized. The results were structured around copper levels in relation to malaria, and any associations or correlations reported. For the meta-analysis, the effect sizes (standardized mean difference, Hedge’s g) along with their 95% confidence intervals (CIs) were computed from the included studies. To account for potential heterogeneity across studies, a random-effects model was used [25]. The *I*^2^ statistic was employed to assess the heterogeneity among the studies, with an *I*^2^ of 0–25%, 25–50%, 50–75%, and 75–100% indicating low, moderate, substantial, and considerable heterogeneity, respectively [26]. To explore the potential sources of heterogeneity, including differences in publication year, study design, study location, or population characteristics, subgroup analyses were conducted. A funnel plot and Egger’s test were used to evaluate publication bias. All statistical analyses were performed using Stata software version 18.0 (StataCorp, College Station, TX, USA).

### 2.8. Sensitivity Analysis

To assess the robustness of the meta-analysis findings, a sensitivity analysis was conducted by sequentially excluding one study at a time and examining the influence of each study on the overall effect size [27].

## 3. Results

### 3.1. Search Results

**Figure 1:**
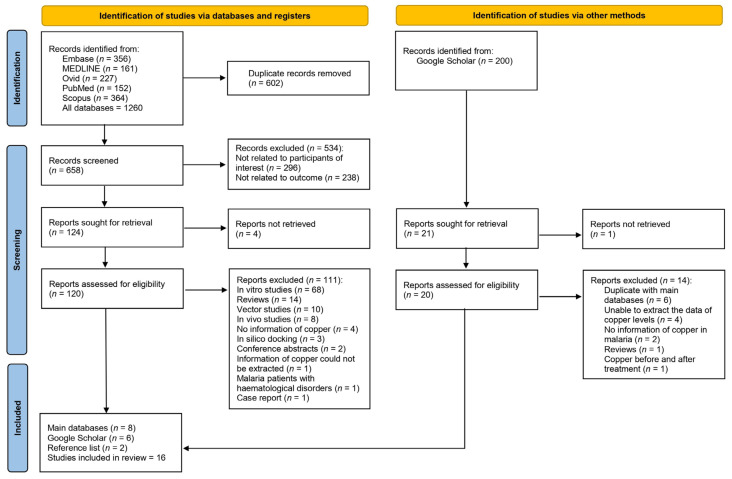
Study flow diagram showing study selection processes.

Database searches identified a total of 1260 records. Specifically, 356, 161, 227, 152, and 364 records were identified from Embase, MEDLINE, Ovid, PubMed, and Scopus, respectively. After the initial search yielded 1260 records, 602 duplicates were removed, leaving 658 for screening. Of these, 534 were excluded (296 for not being related to the participants of interest and 238 for not being related to the outcome), and 124 were earmarked for retrieval but 4 could not be retrieved. Of the 120 records assessed for eligibility, 111 were excluded owing to various criteria, including being in vitro studies or reviews, or lacking pertinent information. After excluding the aforementioned records, eight studies met the eligibility criteria [12,28,29,30,31,32,33,34]. In addition to the main databases, additional records were identified through Google Scholar (*n* = 6) [35,36,37,38,39,40,41] and the reference lists of relevant articles (*n* = 2) [42,43]. Finally, 16 studies were included in the review [12,28,29,30,31,32,33,34,35,37,38,39,40,41,42,43] (Figure 1).

### 3.2. Studies’ Characteristics

The characteristics of the 16 studies included in this review are presented in Table 1. The majority (81.2%) of these studies were published between 2010 and 2023, whereas 18.8% were published between 2000 and 2009. Regarding study designs, 75.0% and 25.0% were cross-sectional and case–control studies, respectively. Geographically, most of the studies (68.8%) were conducted in Africa, with Nigeria being the most represented country (50%). Additionally, studies were conducted in the Cote d’Ivoire (12.5%) and Sudan (6.25%). Asia accounted for 31.2% of the studies, with India (18.8%), Pakistan (6.25%), and Turkey (6.25%) being the locations involved. Regarding the *Plasmodium* species under investigation, 62.5%, 6.25%, and 6.25% of the included studies focused on *P. falciparum*, both *P. falciparum* and *P. vivax*, and *P. vivax* alone, respectively. The *Plasmodium* species was not specified in 25.0% of the studies. The participant groups in these studies varied, with children comprising 43.8% of participants, adults 25.0%, and all age groups 25.0%. One study (6.25%) did not specify the participants’ age groups. Regarding methods for malaria detection, microscopy was predominantly used (93.8%), whereas one study (6.25%) used both microscopy and RDT. Regarding copper measurement, atomic absorption spectrophotometry was used in 81.3% of the studies, and spectrophotometry in 18.7%. Furthermore, serum was used in 75.0% of the studies for copper measurement, whereas plasma was used in 25.0%. Details of the studies are given in Table S2.

### 3.3. Quality of the Studies

Using the JBI critical appraisal checklist for cross-sectional studies, it was observed that most studies (including [12,28]), effectively adhered to key quality criteria, including clear inclusion rules, detailed descriptions of study participants, and valid and reliable methods for measuring exposure and outcomes, supported by objective criteria and proper statistical analyses. However, some studies [30,35] presented vague identification of confounding variables and a lack of clear strategies for addressing these factors. When assessed using the JBI critical appraisal checklist for case–control studies, four studies [31,33,34,38] demonstrated strong methodologies in terms of ensuring group comparability, appropriate case–control matching, and standardized exposure measurements. However, the lack of thorough consideration of confounding factors and unclear information regarding the adequacy of the exposure period are common shortcomings in these studies (Table S3).

### 3.4. Qualitative Synthesis of Copper Levels in Malaria

Most of the studies (50.0%) [29,31,32,33,34,38,42,43] reported that individuals with malaria had significantly higher copper levels than uninfected controls. Among these, Jimoh et al. (2022) [38] and Saad et al. (2013) [32] additionally reported no significant correlation between copper levels and parasite density. Five studies [12,28,30,39,41] indicated that individuals with malaria had significantly lower copper levels than uninfected controls. Furthermore, Ayede et al. (2018) [28] reported a negative correlation between serum copper levels and parasite density. Some studies [35,37] observed no significant difference in copper levels between individuals with malaria and uninfected controls. Moreover, they reported no significant correlation between copper levels and parasite density.

### 3.5. Difference in Copper Levels between Patients with Malaria and Uninfected Controls

**Figure 2:**
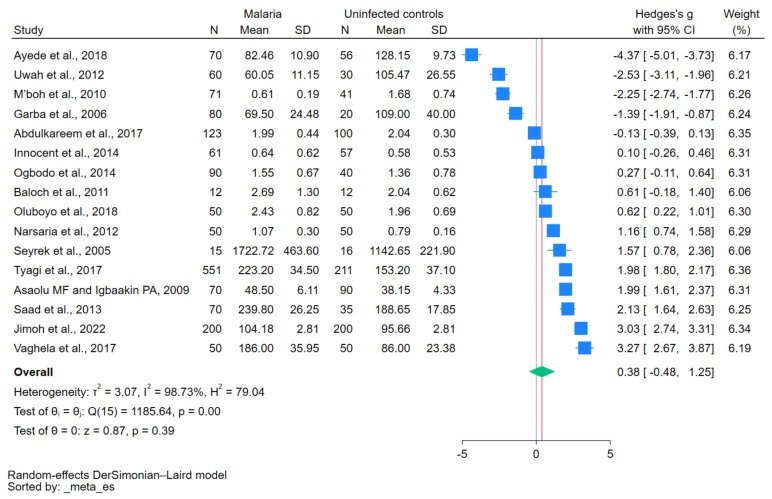
Forest plot showing the difference in copper levels between patients with malaria and uninfected controls. Abbreviations: CI, confidence interval; N, number of participants; SD, standard deviation. Symbols: blue square, effect estimate; green diamond, pooled effect estimate. References: [12,28,29,30,31,32,33,34,35,37,38,39,40,41,42,43].

The difference in copper levels between patients with malaria and uninfected controls was calculated using data from 16 studies [12,28,29,30,31,32,33,34,35,37,38,39,40,41,42,43]. The results showed no difference in copper levels between the two participant groups (*p* = 0.39; Hedges’ g, 0.38; 95% CI, −0.48 to 1.25; *I*^2^, 98.73%; 16 studies, Figure 2). Meta-regression analyses showed that the study design and continent affected the pooled estimate (*p* < 0.05), whereas the remaining covariates showed no significant effects on the pooled estimate, including country, age group, *Plasmodium* species, diagnostic method for malaria, methods for measuring copper levels, and blood samples for copper level measurement (*p* > 0.05).

The subgroup analysis performed based on the publication year from 2010 to 2023 showed no significant difference in copper levels between patients with malaria and uninfected controls (*p* = 0.54; Hedges’ g, 0.31; 95% CI, −0.68 to 1.29; *I*^2^, 98.89%; 13 studies). Similarly, no significant difference in copper levels was noted between patients with malaria and uninfected controls based on the publication year from 2000 to 2009 (*p* = 0.54; Hedges’ g, 0.72; 95% CI, −1.56 to 3.00; *I*^2^, 98.15%; three studies; Table 2). In the subgroup analysis of studies employing a cross-sectional design, no significant difference in copper levels was observed between patients with malaria and uninfected controls (*p* = 0.79; Hedges’ g, −0.14; 95% CI, −1.13 to 0.85; *I*^2^, 98.36%; 12 studies). Conversely, in the subgroup analysis comprising case–control studies, a significant difference in copper levels was noted between patients with malaria and uninfected controls (*p* < 0.01; Hedges’ g, 1.96; 95% CI, 1.19–2.74; *I*^2^, 95.19%; four studies).

When the studies conducted in Africa were analyzed, no significant difference in copper levels was observed between patients with malaria and uninfected controls (*p* = 0.71; Hedges’ g, −0.22; 95% CI, −1.37 to 0.93; *I*^2^, 98.93%; 11 studies). Interestingly, the subgroup analysis of studies conducted in Asia demonstrated a significant difference in copper levels between patients with malaria and uninfected controls (*p* < 0.01; Hedges’ g, 1.74; 95% CI, 1.04 to 2.44; *I*^2^, 90.88%; five studies). In the age-based subgroup analysis, no significant difference in copper levels was observed between children (*p* = 0.95; Hedges’ g, 0.07; 95% CI, −2.04 to 2.18; *I*^2^, 99.30%; seven studies) and adults (*p* = 0.55; Hedges’ g, 0.38; 95% CI, −0.87 to 1.62; *I*^2^, 97.28%; four studies).

In the subgroup analysis of studies focusing on *P. falciparum*, no significant difference in copper levels was noted between patients with malaria and uninfected controls (*p* = 0.74; Hedges’ g, −0.22; 95% CI, −1.52 to 1.08; *I*^2^, 99.04%; 10 studies). For the subgroup that utilized serum blood samples, no significant difference in copper levels was noted between patients with malaria and uninfected controls (*p* = 0.91; Hedges’ g, 0.07; 95% CI, −1.10 to 1.24; *I*^2^, 98.92%; 12 studies), whereas a significant difference was detected in the subgroup employing plasma blood samples (*p* < 0.01; Hedges’ g, 1.33; 95% CI, 0.60 to 2.07; *I*^2^, 93.11%; four studies). When analyzing the subgroup of studies that utilized atomic absorption spectrophotometry for copper measurement, no significant difference in copper levels was observed between patients with malaria and uninfected controls (*p* = 0.59; Hedges’ g, 0.26; 95% CI, −0.70 to 1.22; *I*^2^, 98.56%; 13 studies). When analyzing the subgroup of studies from different databases, no significant difference in copper levels was observed between patients with malaria and uninfected controls among studies from the main databases (*p* = 0.75; Hedges’ g, 0.20; 95% CI, −1.05 to 1.45; *I*^2^, 98.72%; eight studies) and other sources (*p* = 0.42; Hedges’ g, 0.56; 95% CI, −0.80 to 0.93; *I*^2^, 98.90%; eight studies; Table 2).

### 3.6. Publication Bias

**Figure 3:**
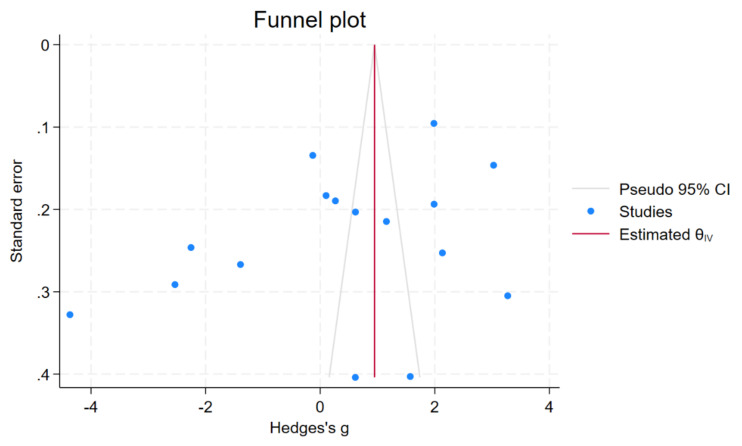
Funnel plot showing an asymmetrical distribution of the effect estimate of copper levels between patients with malaria and uninfected controls. Abbreviation: CI, confidence interval.

A funnel plot and Egger’s test were used to assess the presence of publication bias. The funnel plot exhibited asymmetry (Figure 3); however, Egger’s test did not show significant small-study effects (*p* = 0.22). To further investigate potential publication bias, a non-parametric trim-and-fill analysis was performed. The results indicated no significant difference in copper levels between patients with malaria and uninfected controls (Hedges’ g, 0.38; 95% CI, −0.48 to 1.25; 16 studies). These findings suggest that the observed asymmetry in the funnel plot is attributed to other factors, such as heterogeneity, rather than the absence of small studies in the meta-analysis.

### 3.7. Sensitivity Analysis

**Figure 4:**
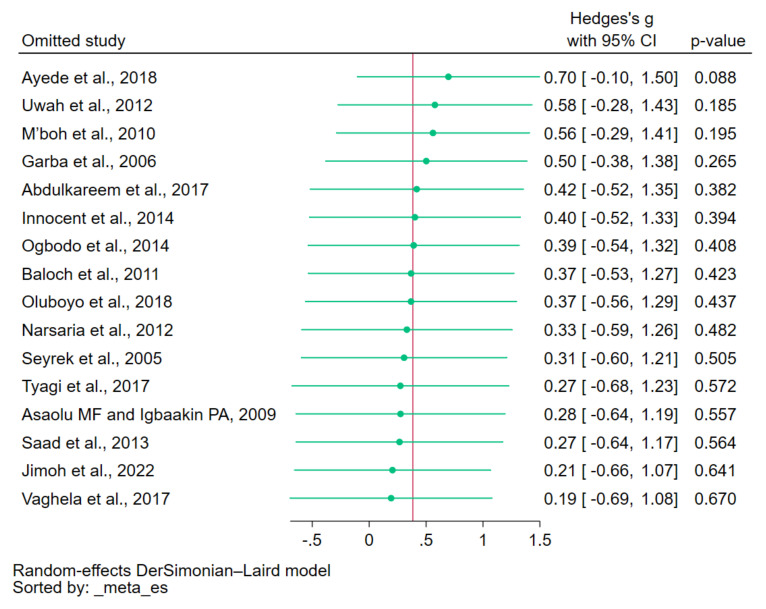
Leave-one-out method showing an outlier in the meta-analysis of the difference in copper levels between patients with malaria and uninfected controls. Abbreviation: CI, confidence interval. Symbols: green dot, pooled effect estimate of each re-run analysis; green line, confidence interval; red line, pooled effect estimate of all re-run analyses. References: [12,28,29,30,31,32,33,34,35,37,38,39,40,41,42,43].

To assess the influence of individual studies on the pooled effect estimate, a leave-one-out meta-analysis was conducted. The results demonstrated that upon excluding each study from the re-run meta-analyses, none of the 15 re-run analyses showed a non-significant difference in copper levels between patients with malaria and uninfected controls (*p* > 0.05, Figure 4). These findings indicate that the overall results of the meta-analysis remained relatively stable and robust.

## 4. Discussion

This systematic review showed that most of the included studies reported that individuals with malaria had significantly higher copper levels than uninfected controls. Nevertheless, a meta-analysis using data from 16 studies indicated no significant difference in copper levels between the two participant groups. The overall findings suggested that *Plasmodium* infections did not alter the blood copper level. The high heterogeneity among the included studies must be noted, as evidenced by the high *I*^2^ value (98.73%). Of note, two studies within this majority group, Jimoh et al. [38] and Saad et al. [32], reported no significant correlation between copper levels and parasite density. This suggests that copper levels are not directly influenced by the severity of the infection in these cases. Conversely, five studies indicated that individuals with malaria had significantly lower copper levels than uninfected controls [12,28,30,39,41]. Additionally, Ayede et al. [28] reported a negative correlation between serum copper levels and parasite density. These findings suggest a contrasting relationship between copper levels and malaria, suggesting that lower copper levels are associated with the presence or severity of infection. Some studies reported no significant difference in copper levels between individuals with malaria and uninfected controls [35,37]. These findings suggest that copper levels are not directly influenced by the presence of malaria in these particular cases, and there are other factors to consider.

The high heterogeneity among the included studies in this meta-analysis, as evidenced by the high *I*^2^ value, was explored using meta-regression and subgroup analyses. To investigate various factors that could potentially affect the results, subgroup analyses were performed. The results showed that publication years, age ranges, *Plasmodium* species, and the method of copper measurement did not affect the meta-analysis results. These findings suggest that these factors are not significant in determining copper levels in individuals with malaria. In contrast, when the studies were stratified by continent, the analysis showed interesting results. In studies conducted in Africa, no significant difference in copper levels was observed between patients with malaria and uninfected controls. However, in studies conducted in Asia, a significant difference was noted, suggesting potential geographical variation in copper levels among malaria cases. The observed higher copper levels in patients with malaria than those in uninfected controls, as reported by studies conducted in Asia, were not fully elucidated. Several potential explanations are proposed. One possibility is that individuals with malaria may concurrently have other conditions, such as Wilson’s disease. This disease, which is more prevalent in the Asian population than in other populations, is associated with elevated copper levels [44,45,46]. Another possibility is that there is a difference in immune responses to malaria between the Asian and African populations. Early exposure to malaria in holoendemic settings, including Africa, may protect the population by enabling them to develop naturally acquired immunity to *P*. *falciparum* malaria [47]. This immunity may help sustain copper levels, considering that copper is essential for the immune system, particularly for neutrophil function and superoxide anion generation to eliminate pathogens [21]. In African children, immune responses may be lacking, potentially leading to lower serum copper levels. In contrast, most *Plasmodium* infections in Asia occur in adults. Consequently, this population may have a stronger immune response against malaria than African children, potentially leading to elevated copper levels. Another possibility is that the Asian population may have higher incidences of conditions such as gestational diabetes mellitus [48], preeclampsia [49], and heart failure [50], all of which can contribute to elevated blood copper levels.

Furthermore, the blood samples used for copper level measurement yielded interesting results. The subgroup analysis based on blood sample type showed no significant difference when serum samples were utilized. However, a significant difference was observed when plasma samples were employed, suggesting that the choice of blood sample type influences the measured copper levels. Copper in the blood is very essential as it contributes to the immune response of the host to the antigenic challenge by malaria parasites since both elements are important for normal immune function [32]. The increase in copper levels observed in malaria infections may be attributed to the following two factors: the body’s immune response to combat the malaria parasite or zinc absorption inhibition from the intestine [38].

The inconsistency regarding copper level alterations in malaria may be because of its dual role, including elevated copper levels resulting in increased oxidative damage to lipids, proteins, and DNA, and copper deficiency that is associated with oxidative stress. For the first role, copper plays a crucial role in stimulating the immune system to combat infections, support tissue repair, and promote healing. Furthermore, the significant increase in copper levels may be a result of the body’s attempt to counteract free radical production, as malaria is characterized by inflammation and oxidative stress. The higher copper levels observed may be a response that occurs to enhance the activity of antioxidant enzymes, thereby addressing the increased free radical generation occurring during malaria infection. Saad et al. reported an increase in serum copper levels, which is attributed to its role as a component of ceruloplasmin, an acute phase reactant and antioxidant [32]. For the second role, copper plays a protective function by enhancing the activity of enzymes, including superoxide dismutase, thereby safeguarding the body against the potential detrimental effects of free radicals [38]. Consequently, the reduced copper levels observed in previous studies could be attributed to the scavenging of oxidants released by immune cells, consequently protecting the host and mitigating the oxidative impact of parasitemia [28]. Therefore, the decrease in copper levels in patients with malaria may be because of the increased copper utilization for oxidant scavenging during *Plasmodium* infection. A decrease in copper levels in patients with malaria may be observed more frequently in males than that in females, as males normally have low copper levels regardless of the presence of malaria infection [51].

The findings regarding copper level alterations in malaria demonstrate certain limitations and considerations. While most of the reviewed studies reported significantly higher copper levels in individuals with malaria than those in uninfected controls, the meta-analysis results showed no significant overall difference. This inconsistency may be attributed to the high heterogeneity among the included studies, as indicated by the high *I*^2^ value. These limitations highlight the need for more comprehensive and standardized studies to elucidate the role of copper in malaria. Addressing the heterogeneity, considering geographical factors and the blood samples used for measuring copper levels will contribute to a better understanding of the mechanisms underlying copper alterations in malaria.

## 5. Conclusions

The qualitative synthesis of the reviewed studies suggests a complex relationship between copper levels and malaria. While some studies noted higher copper levels in individuals with malaria, others observed lower levels. The meta-analysis results showed no significant difference in copper levels between patients with malaria and uninfected controls overall. These findings highlight the need for further studies to better understand the role of copper in malaria pathogenesis by considering geographical factors and the blood sample type used for copper level measurement.

## Figures and Tables

**Table 1:** Characteristics of included studies.

Characteristics	*N* (16 Studies)	%
Publication year		
2010–2023	13	81.2
2000–2009	3	18.8
Study designs		
Cross-sectional studies	12	75.0
Case–control studies	4	25.0
Study areas		
Africa	11	68.8
Nigeria	8	50.0
Cote d’Ivoire	2	12.5
Sudan	1	6.25
Asia	5	31.2
India	3	18.8
Pakistan	1	6.25
Turkey	1	6.25
*Plasmodium* species		
*P. falciparum*	10	62.5
*P. falciparum* and *P. vivax*	1	6.25
*P. vivax*	1	6.25
Not specified	4	25.0
Age group		
Children	7	43.8
Adults	4	25.0
All age groups	4	25.0
Not specified	1	6.25
Methods for malaria detection		
Microscopy	15	93.8
Microscopy/RDT	1	6.25
Methods for copper measurement		
Atomic absorption spectrophotometry	13	81.3
Spectrophotometry	3	18.7
Blood used for copper measurement		
Serum	12	75.0
Plasma	4	25.0

RDT, rapid diagnostic test.

**Table 2:** Subgroup analyses of copper levels between malaria cases and uninfected controls.

Subgroup Analyses	*p* Value	Hedges’ g (95% CI)	*I*^2^ (%)	Number of Studies
Publication years				
2010–2023	0.54	0.31 (−0.68 to 1.29)	98.89	13
2000–2009	0.54	0.72 (−1.56 to 3.00)	98.15	3
Study design				
Cross–sectional study	0.79	−0.14 (−1.13 to 0.85)	98.36	12
Case–control study	<0.01	1.96 (1.19 to 2.74)	95.19	4
Continent				
Africa	0.71	−0.22 (−1.37 to 0.93)	98.93	11
Asia	<0.01	1.74 (1.04 to 2.44)	90.88	5
Age group				
Children	0.95	0.07 (−2.04 to 2.18)	99.30	7
Adults	0.55	0.38 (−0.87 to 1.62)	97.28	4
All age groups	0.18	0.87 (−0.40 to 2.14)	98.50	4
Age not specified	N/A	0.61 (−0.18 to 1.40)	N/A	1
*Plasmodium* species				
*P. falciparum*	0.74	−0.22 (−1.52 to 1.08)	99.04	10
*P. vivax*	N/A	1.57 (0.78 to 2.36)	N/A	1
*P. falciparum*, *P. vivax*	N/A	3.27 (2.67 to 3.87)	N/A	1
Not specified	0.09	0.88 (0.13 to 1.90)	96.72	4
Diagnostic method for malaria				
Microscopy	0.48	0.33 (−0.59 to 1.26)	98.82	15
Microscopy/RDT	N/A	1.16 (0.74 to 1.58)	N/A	1
Types of blood samples				
Serum	0.91	0.07 (−1.10 to 1.24)	98.92	12
Plasma	<0.01	1.33 (0.60 to 2.07)	93.11	4
Methods for copper measurement				
Atomic absorption spectrophotometry	0.59	0.26 (−0.70 to 1.22)	98.56	13
Spectrophotometry	0.53	0.91 (−1.93 to 3.75)	99.19	3
Databases				
Main databases	0.75	0.20 (−1.05 to 1.45)	98.72	8
Other sources (Google Scholar and reference list)	0.42	0.56 (−0.80 to 1.93)	98.90	8

Abbreviations: CI, confidence interval; N/A, not assessed; RDT, rapid diagnostic test.

## Data Availability

All data relating to the present study are available in this manuscript and the Supplementary Materials.

## Supplementary Materials

The following supporting information can be downloaded at: https://www.mdpi.com/article/10.3390/nu15224749/s1, Table S1: Search terms; Table S2: Details of the included studies; Table S3: Quality of included studies; Table S4: Meta-regression results.
