# Supplementary material for: A Systematic Review and Meta-Analysis Exploring Variations in Copper Levels between Individuals with Malaria and Uninfected Controls
Source: Nutrients. 2023 Nov 10;15(22):4749. doi: 10.3390/nu15224749 (PMC10675583; doi:10.3390/nu15224749)
Supplement: Supplementary file 1 [file nutrients-15-04749-s001.zip › Table S1. Search terms.pdf]

Table S1. Search terms.

General keywords

copper AND (malaria OR plasmodium OR “Plasmodium Infection“ OR “Remittent Fever“ OR “Marsh Fever“ OR Paludism)

PubMed 21 June 2023

No.	Key concept	Search terms	Results
1.	Copper	copper[Text Word] OR copper[MeSH Terms]	156,137
2.	Malaria	malaria[Text Word] OR plasmodium[Text Word] OR “Infections, Plasmodium“[MeSH Terms] OR “Infection, Plasmodium“[MeSH Terms] OR “Plasmodium Infection“[MeSH Terms] OR “Remittent Fever“[MeSH Terms] OR “Fever, Remittent“[MeSH Terms] OR “Marsh Fever“[MeSH Terms] OR “Fever, Marsh“[MeSH Terms] OR Paludism [MeSH Terms]	118,242
3.	1 AND 2	(copper[Text Word] OR copper[MeSH Terms]) AND (malaria[Text Word] OR plasmodium[Text Word] OR “Infections, Plasmodium“[MeSH Terms] OR “Infection, Plasmodium“[MeSH Terms] OR “Plasmodium Infection“[MeSH Terms] OR “Remittent Fever“[MeSH Terms] OR “Fever, Remittent“[MeSH Terms] OR “Marsh Fever“[MeSH Terms] OR “Fever, Marsh“[MeSH Terms] OR Paludism [MeSH Terms])	152

Embase 21 June 2023

No.	Key concept	Search terms	Results
1.	Copper	copper:ti,ab,kw,de OR copper/exp	245,110
2.	Malaria	malaria:ti,ab,kw,de OR plasmodium:ti,ab,kw,de OR ‘Remittent Fever’:ti,ab,kw,de OR ‘Marsh Fever’:ti,ab,kw,de OR Paludism:ti,ab,kw,de OR malaria/exp	156,345
3.	1 AND 2	(copper:ti,ab,kw,de OR copper/exp) AND (malaria:ti,ab,kw,de OR plasmodium:ti,ab,kw,de OR ‘Remittent Fever’:ti,ab,kw,de OR ‘Marsh Fever’:ti,ab,kw,de OR Paludism:ti,ab,kw,de OR malaria/exp)	356

Scopus 21 June 2023

No.	Key concept	Search terms	Results

1.	Copper	TITLE-ABS-KEY (copper)	849,374
2.	Malaria	TITLE-ABS-KEY ((malaria OR plasmodium OR "plasmodium infection" OR "remittent fever" OR "marsh fever" OR paludism))	157,025
3.	1 AND 2	TITLE-ABS-KEY (copper) AND (TITLE-ABS-KEY ((malaria OR plasmodium OR "Plasmodium Infection" OR "Remittent Fever" OR "Marsh Fever" OR paludism)))	364

MEDLINE 21 June 2023

No.	Key concept	Search terms	Results
1.	Copper AND Malaria	copper AND (malaria OR plasmodium OR “Plasmodium Infection“ OR “Remittent Fever“ OR “Marsh Fever“ OR Paludism)	161

Ovid 21 June 2023

No.	Key concept	Search terms	Results
1.	Copper AND Malaria	copper AND (malaria OR plasmodium OR “Plasmodium Infection“ OR “Remittent Fever“ OR “Marsh Fever“ OR Paludism) {Including Limited Related Terms}limit to (ovid full text available and articles with abstracts and original articles)	227
