# Supplementary material for: A Systematic Review and Meta-Analysis Exploring Variations in Copper Levels between Individuals with Malaria and Uninfected Controls
Source: Nutrients. 2023 Nov 10;15(22):4749. doi: 10.3390/nu15224749 (PMC10675583; doi:10.3390/nu15224749)
Supplement: Supplementary file 1 [file nutrients-15-04749-s001.zip › Table S4. Meta-regression results.pdf]

Table S4. Meta-regression results

**Meta-analysis of copper**	**Covariates**	***P* value**	**tau2**	**I^2 (%)**	**R-squared (%)**	
**Malaria cases vs uninfected controls**	Publication years	0.725	3.274	98.82	0	16
	Study design	0.017	2.218	98.09	27.67	16
	Country	0.265	3.303	98.83	0	16
	Continent	0.041	3.027	98.56	1.29	16
	Age group	0.934	3.914	98.97	0	16
	Plasmodium species	0.248	3.373	98.84	0	16
	Diagnostic method for malaria	0.661	3.265	98.82	0	16
	Method for measurement of copper	0.587	3.472	98.71	0	16
	Blood samples	0.230	3.272	98.68	0	16
